# A Model Curriculum for a Helicopter Emergency Medicine Services (HEMS) Rotation for Resident Physicians

**DOI:** 10.21980/J8GP97

**Published:** 2020-07-15

**Authors:** Jordan Imoehl, Michael T Steuerwald, Andrew D Cathers

**Affiliations:** * University of Wisconsin-Madison School of Medicine and Public Health, Department of Emergency Medicine, Madison, WI; ^ UW Med Flight, Madison, WI

## Abstract

**Audience:**

This curriculum is designed for resident physicians at all levels of training who have an interest in HEMS.

**Length of Curriculum:**

This curriculum is designed to run over a 28-day period.

**Introduction:**

Helicopter emergency medicine services play a critical role in patient transport, and resident physicians may often encounter patients transported by HEMS programs. Residents, and emergency medicine residents in particular, are being offered more opportunities to gain flight experience with HEMS programs; however, these experiences may be highly variable. These inconsistencies in training experiences may lead to incomplete understanding of HEMS systems and patient care performed during flight.

**Educational Goals:**

The primary objective of this course is to present a standardized curriculum which allows learners to gain understanding of HEMS systems and retrieval medicine while practicing safety in the aviation environment.

**Educational Methods:**

The educational strategies used in this curriculum include hands-on training with senior flight staff, asynchronous learning via access to a curated reading “library,” and in-person learning via ride-along experience on the aircraft and program operational meeting attendance.

**Research Methods:**

This curriculum was introduced at the authors’ institution and was completed by 11 rotating resident physicians, primarily PGY-2 and PGY-3 emergency medicine residents. Learners completed free-form feedback forms as well as a numerically graded post rotation survey. Learner feedback was used to identify areas where additional instruction was necessary and make changes to optimize learner flight experience.

**Results:**

The curriculum was graded by learners on a 5-point Likert scale. The statement of “My overall educational experience during the rotation met my expectations and the learning objectives outlined at the beginning of the rotation” received an average score of 4.7 based on 1-Disagree to 5-Completely agree. The statement “The longitudinal curriculum contributed to my learning” averaged 4.5. A score of 4.9 was given for the statement “I found the rotation to be of use in my emergency medicine training.” Free-form feedback was also solicited by learners and included comments such as “*It was helpful for me to spend some time in the airway* [and procedure] *lab and go through the shift topics*.”

**Discussion:**

As evidenced by the learner feedback and survey results, the curriculum was effective in meeting the designed educational objectives, and learner feedback was largely positive in nature. Utilizing dedicated daily teaching topics was key to providing a standardized learning experience and ensuring that education progressed without reliance on flight call volumes.

**Topics:**

Helicopter aviation safety, HEMS crew member operations, retrieval medicine, advanced trauma care, advanced airway management, ventilator management, HEMS program operations.

## USER GUIDE

[Table t1-jetem-5-3-c82]

**Table t1-jetem-5-3-c82:** 

List of Resources:	
Abstract	82
User Guide	84
Curriculum Chart	88
Appendix A: HEMS Program Framework	93
Appendix B: Functions of HEMS Program	95
Appendix C: Safety in HEMS Aviation	98
Appendix D: Learner Shift Expectations	100
Appendix E: Radio Use	102
Appendix F: Advanced Discussion Topics	107
Appendix G: Ventilator Competency	127
Appendix H: HEMS Crew Medical Operations	129
Appendix I: Non-Flight HEMS Program Operations	131

**Learner Audience:**

Interns, Junior Residents, Senior Residents

**Length of Curriculum:**

This curriculum is designed to run over a 28-day period but could be adjusted based on timing needs.

**Topics:**

Helicopter aviation safety, HEMS crew member operations, retrieval medicine, advanced trauma care, advanced airway management, ventilator management, HEMS program operations.

**Objectives:**

The goals of this curriculum are:

1. Learners will show understanding of, and participate in, safe practices in and around the aircraft.
2. Learners will demonstrate understanding of operations as a HEMS crew member as well as having knowledge of HEMS transport protocols.
  - Understand pre-hospital triage and emergency care delivery
  - Practice rapid assessment and stabilization of critically ill and injured patients prior to and during transport
  - Learn to communicate effectively with medical staff from transferring facilities in the coordination of referral patients
  - Learn to communicate effectively with all levels of EMS providers
3. Learners will demonstrate knowledge of advanced topics and procedures relevant to critical care transport and retrieval medicine.
  - Learn the indications and contraindications for commonly used blood products, intubation and sedation medications, and hyperosmolar agents
  - Understand methods for difficult airway management including adjuncts for endotracheal intubation as well as extra-glottic devices and surgical airways
  - Demonstrate appropriate use of a cardiac monitor for defibrillation, cardioversion, and cardiac pacing
  - Learn the approach to managing ventilated patients who are hard to ventilate or develop high peak airway pressures
4. Learners will develop an understanding of the functions of a HEMS program.
  - Develop understanding of appropriate utilization of ground and air medical transport
  - Explore the integration of ground-based EMS and helicopter EMS into a unified system of pre-hospital and inter-hospital providers
5. Learners will understand a HEMS program from an operations perspective with a focus on EMS quality improvement

### Brief introduction

There are roughly 400,000 patients transported by helicopter emergency medical services (HEMS) annually in the US.^1^ During residency training, resident physicians often encounter critically ill and injured patients, as well as patients with time sensitive diagnoses, whom are transferred via HEMS. While the ACMGE requires all emergency medicine residents receive training in emergency medical services,^2^ no specific training in HEMS is required and many trainees may be unaware of the intricacies of HEMS operations.

Resident physician HEMS participation is becoming more common among emergency medicine residency programs,^3^ and the experiences of residents participating in HEMS rotations can be highly variable.^4^ Despite increasing resident HEMS participation and continued calls for action regarding the necessity of resident curricula, only 36% of residency programs surveyed had a formal HEMS curriculum in place.^5^ The focus and depth of these curricula can be highly variable.^6^,^7^

We present a curriculum designed to allow learners the opportunity to learn advanced retrieval medicine, practice safety in the aviation environment, and observe the management functions of a HEMS operator. This program is designed to be replicated and adapted by a variety of emergency residency programs. Ideally this would allow programs without any formal HEMS curriculum to establish one and allow for better standardization of HEMS training across emergency medicine residency programs.

### Problem identification, general and targeted needs assessment

There is currently no standardized HEMS curriculum in use by emergency medicine residency programs despite increasing involvement of resident physicians with HEMS. More concerning is that some training programs have no formal established curriculum, causing variability in experiences among learners. Ultimately this may have consequences on patient care and resident understanding of HEMS operations.

### Goals of the curriculum

The aim of this curriculum is to establish a structured, standardized model for residency programs offering HEMS experience to residents.

### Objectives of the curriculum

1. Learners will show understanding of, and participate in, safe practices in and around the aircraft.
2. Learners will demonstrate understanding of operations as a HEMS crew member as well as having knowledge of HEMS transport protocols.
  - Understand pre-hospital triage and emergency care delivery
  - Practice rapid assessment and stabilization of critically ill and injured patients prior to and during transport
  - Learn to communicate effectively with medical staff from transferring facilities in the coordination of referral patients
  - Learn to communicate effectively with all levels of EMS providers
3. Learners will demonstrate knowledge of advanced topics and procedures relevant to critical care transport and retrieval medicine.
  - Learn the indications and contraindications for commonly used blood products, intubation and sedation medications, and hyperosmolar agents
  - Understand methods for difficult airway management including adjuncts for endotracheal intubation as well as extra-glottic devices and surgical airways
  - Demonstrate appropriate use of a cardiac monitor for defibrillation, cardioversion, and cardiac pacing
  - Learn the approach to managing ventilated patients who are hard to ventilate or develop high peak airway pressures
4. Learners will develop an understanding of the functions of a HEMS program.
  - Develop understanding of appropriate utilization of ground and air medical transport
  - Explore the integration of ground-based EMS and helicopter EMS into a unified system of pre-hospital and inter-hospital providers
5. Learners will understand a HEMS program from an operations perspective with a focus on EMS quality improvement

### Educational strategies

(See curriculum chart)

### Results and tips for successful implementation

Our institution has a long history of offering emergency medicine residents flight opportunities, and our curriculum had historically been an organically grown process. However, the most recent iteration presented here went into effect in 2018. For programs looking to implement this curriculum, we would recommend instating this as a complete bundle because piecemeal implementation may lead to inconsistent experiences by learners.

Many learners have limited aviation experience, and safety should be a top priority during training. Residents should be outfitted with necessary equipment for safe flight operation (flight suit, helmets, boots, etc.) via either personal or program supplies. Learners should also feel comfortable working around the aircraft and demonstrate safe operations to the pilot or a crew member prior to beginning flight experiences.

Prior to flight, learners should also be given instruction in basic flight crew tasks such as usage of radios, loading and unloading of the stretcher/litter, and usage of the cardiac monitor with the intention of functioning as a full member of the team. They should be given access to any available protocols which guide team performance. Learners will attend all crew and program briefings and be an integrated member of the team.

Learners will gain clinical knowledge via ride-along experiences with the flight crew. At our institution, learners are scheduled for twelve, 12-hour shifts. Flight experience will include assessment and stabilization of critically ill and injured patients in the prehospital setting, as well as the continued care of critically ill patients during intra-facility transport. Learners should be encouraged to be involved with patient care to the level of training of the learner and within comfort of the HEMS crew. Involvement in patient care will also require interfacing with ground-based EMS in pre-hospital settings and hospital medical staff during intra-facility transports. A HIPAA compliant case log should be maintained to aid with case review and with tracking flight experience. Learners will be given feedback at the end of each shift by means of a dedicated post-shift feedback card which is filled out by the medical staff they are working with that day.

It is hoped that during these flight experiences learners will be exposed to a variety of critical care circumstances including trauma, cardiac, neurologic, and airway emergencies. However, daily topics covering these subjects are provided for discussion because there may be variability in the flight experiences between learners. If possible, training equipment for flight staff should also be made available to learners to gain hands on experience. At our institution this includes pediatric and adult airway mannequins for practicing airway management strategies. A ventilator with test lung is available for teaching and practice with ventilatory modes and strategies. Access to hemorrhage control devices such as tourniquets, pressure dressings, and combat gauze also provide practical experience. Learners are also provided access to a reading “library” which contains texts and literature relevant to the practice of transport medicine.^8^–^15^

Learners can gain an appreciation for the non-flight operations of a HEMS program by observing management meetings. In our program we include learners in the following meetings:

1. Education and Training, which focuses on planning didactics and continuing education for critical care transport (CCT) staff
2. Performance Improvement, which focuses on performance improvement activities including case review and utilization management
3. Operations, which focuses on implementation of policies, workflows, and practices related to clinical care
4. Quality and Safety, which focuses on case review, improving the quality of clinical care, and reviewing safety incidents within our program and the aeromedical transport industry
5. Morbidity and Mortality, which focuses on an in-depth review of transports performed by the program which involved extraordinary circumstances or learning opportunities for CCT staff
6. All Staff Meeting, which focuses on dissemination of any critical information to all employed staff members and allows for staff feedback on changes or issues which have arisen

### Evaluation and feedback

Eleven learners have successfully completed the curriculum as presented in this publication. The curriculum was graded by learners on a 5-point Likert scale survey with “1” indicating learners disagree with the given statement and “5” indicating the learner is in complete agreement. It received an average score of 4.7 regarding the statement, “My overall educational experience during the Med Flight rotation met my expectations and the learning objectives outlined at the beginning of the rotation.” The statement, “The longitudinal curriculum contributed to my learning,” scored 4.5 based on the same scale. A score of 4.9 was given for the statement “I found the rotation to be of use in my emergency medicine training.” Feedback was also obtained via free-form feedback responses requested from individual learners upon completion of the curriculum, and included comments such as, “*It was helpful for me to spend some time in the airway* [and procedure] *lab and go through the shift topics*.” As evidenced by the survey and individual feedback, learners clearly found value in the rotation and felt the curriculum helped contribute to rotator learning.

The curriculum also underwent review by our continuous residency improvement committee.^16^ These feedback mechanisms have led to the streamlining of some of the onboarding process. Learners are also now made aware of program meeting dates and times well in advance and prior to scheduling their shifts. Residents are now also offered the opportunity to complete shifts at our remote bases where they may have opportunities for higher flight volumes.

## Figures and Tables

**Table t2-jetem-5-3-c82:** 

Topic	Recommended Educational Strategy	Educational Content	Objectives	Learners	Timing, Resources Needed	Recommended Assessment, Milestones Addressed
Introduction to HEMS	Review of provided documents. In person discussion with flight crew and HEMS program staff.	- Tour of facilities - Introduction to transport manager, chief transport nurse, medical director - Introduction to medical crew and pilots - Flight mission profiles - Flight crew configuration - Common aircraft in HEMS aviation - Indications for air transport - Risks and benefits of air transport	Learners should be comfortable with their working environment and co-workers. Learners will demonstrate general knowledge of the flight capabilities of their program.	Intern or resident	2 hours. Instructors: 1 Equipment: Computer, hand radio, medically equipped aircraft. Written supplement is available for review by learners and instructor for flight mission profiles, crew configuration, and common aircraft in use in Appendix A. Risks, benefits, and indications for air transport are discussed in Appendix B.	Assessment: in person demonstration of familiarity with facilities and crew and understanding of programs capabilities.
Safety in HEMS Aviation	Hands-on session in aircraft. Hands-on session in hanger.	- Appropriate sizing of helmet and flight suit - Seatbelt use - Safe practices around rotor blades - Fire extinguisher location and usage - Use of emergency door or window exit - Life vest usage and water ditching procedure - Engine shutdown methods - Emergency Location Transponder Activation - Usage of oxygen shutoff - Usage of rotor brake - Usage of emergency procedures checklists for pilot aid - Usage of emergency fuel shutoff on Helipad - Performing preflight walk around	The learner will be able to safely perform work duties in and around the aircraft and have understanding of appropriate next steps if an in-flight emergency develops.	Intern or Resident	2 hours. Instructors:1 Equipment: aircraft. Appendix C contains written supplements for implementation of safety teachings.	Assessment: In person demonstration of usage proficiency.
Crew Member Operations	Hands-on session in aircraft/hanger. Asynchronous: computerbased review of protocols and supplements. Clinical Experience.	- Access to protocol repository - Review of shift expectations document - Review radio usage - Stretcher loading/unloadi ng - Attend all crew and program briefings.	After adequate training and flight experience, the learner will be able to participate in all crew member operations.	Intern or Resident	2 hours. Instructors: 1 Equipment: Computer, hand radio, medically equipped aircraft. Appendix D contains a sample shift expectations document. Sample radio usage manual is contained in Appendix E.	Assessment: In person demonstration of proficiency.
Advanced topics in critical care transport	In person discussion or hands on session with medical flight crew while on shift. Asynchronous: access to resident reading library.	- TXA and Blood Product Administration - DOPES protocol - SALAD Intubation - Use of the Bougie - High PAP alarms - Ventilation in difficult-tooxygenate patient - RSI Medications and Post- Intubation Sedation/Analg esia - Use of the Monitor for EtCO2, Pacing, and Defibrillation - Management of Elevated ICP, Use of hypertonic saline - Use of Ventilator (SIMV, Pressure Control, NIPPV) - Failed Airway Procedures – EGD and Cricothyrotomy - Application of Pelvic Binder development of ventilator competency	Learner should understand the indications and dosages for medications (TXA, blood products, RSI meds, post intubation sedation and analgesia, hypertonic saline). Learner can demonstrate usage and troubleshooting of pelvic binder, airway devices (ETT, EGDs, bougie, cricothyrotomy kit), ventilator, monitor (with pacing and defib).	Intern or Resident	~30 minutes per topic (12 topics). Instructors: 1 Equipment: None required. Ventilator, airway devices, cardiac monitor, pelvic binder, and splints are optional for additional hands on instruction. Written supplement available for instructor/learner regarding advanced discussion topics (Appendix F) and ventilator competency (Appendix G).	Assessment: in person assessment of understanding by instructor.
Retrieval medicine	Discussion with medical crew members. Clinical experience during ridealong shifts.	- pre-hospital triage and emergency care delivery - rapid assessment and stabilization of critically ill and injured patients prior to and during transport - communication with medical staff from transferring facilities in the coordination of referral patients - communication with all levels of EMS providers.	Learner should take steps to participate in patient care during ride along flights within comfort level of the learner and the transport team.	Intern or Resident	Twelve, 12-hour ride-along shifts. Equipment: flight ready HEMS aircraft. Written supplement is available for learners and instructors to review regarding triage, patient assessment, and communication (Appendix H).	Assessment: End of shift feedback cards. End of rotation review with supervising faculty.
HEMS program management and operations	In person experience via attendance of program meetings.	- Attend Critical Care Transport (CCT) education meeting - Attend CCT performance improvement meeting - Attend CCT operations meeting - Attend CCT nursing education meeting - Attend CCT quality and safety meeting - Attend CCT Morbidity and Mortality (M&M) Conference - Attend all critical care staff monthly meeting.	Learner should gain insight into the planning and execution of HEMS system management.	Intern or Resident	Duration: 1 hour for CCT education meeting. 1.5 hours for CCT performance improvement meeting, quality and safety meeting, and M&M. 2 hours for CCT operations meeting. Written guidance is provided on implementation practices (Appendix I).	Assessment: Documented attendance at meeting.

**Figure 1:**
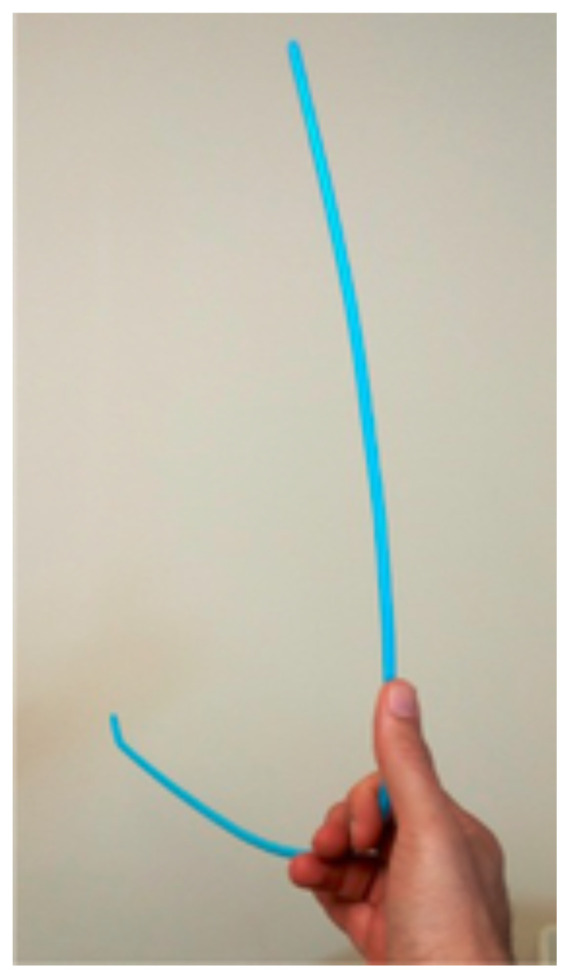
Demonstration of "shaka" grip for a bougie. Image source: author’s own image

**Figure 2:**
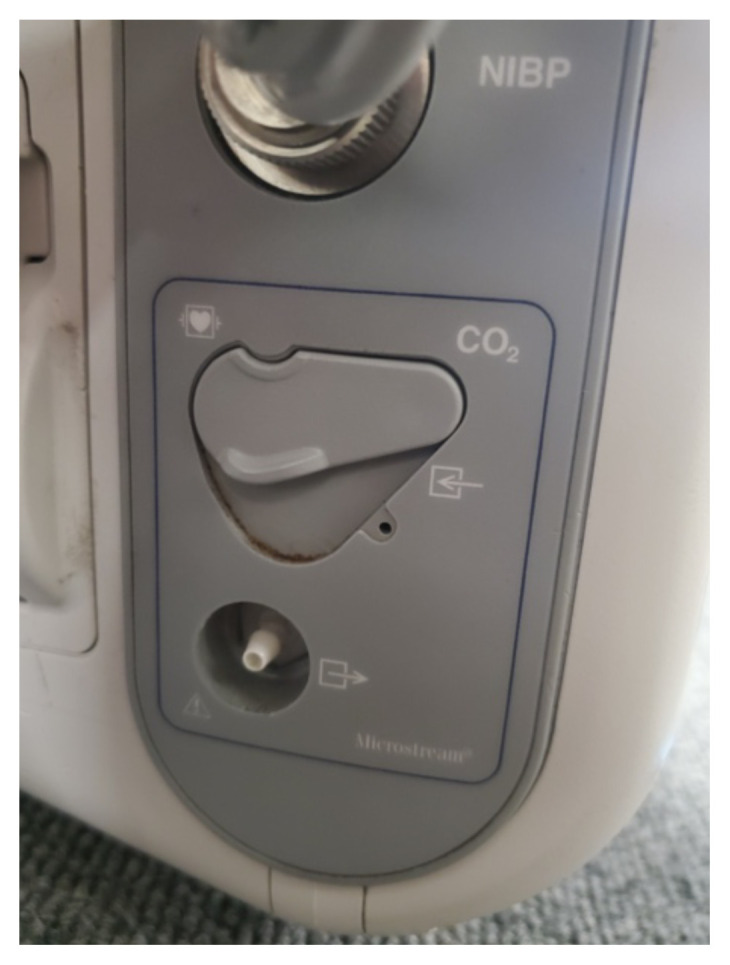
Side view of monitor showing covering of EtCO2 connection Image source: author’s own image

**Figure 3:**
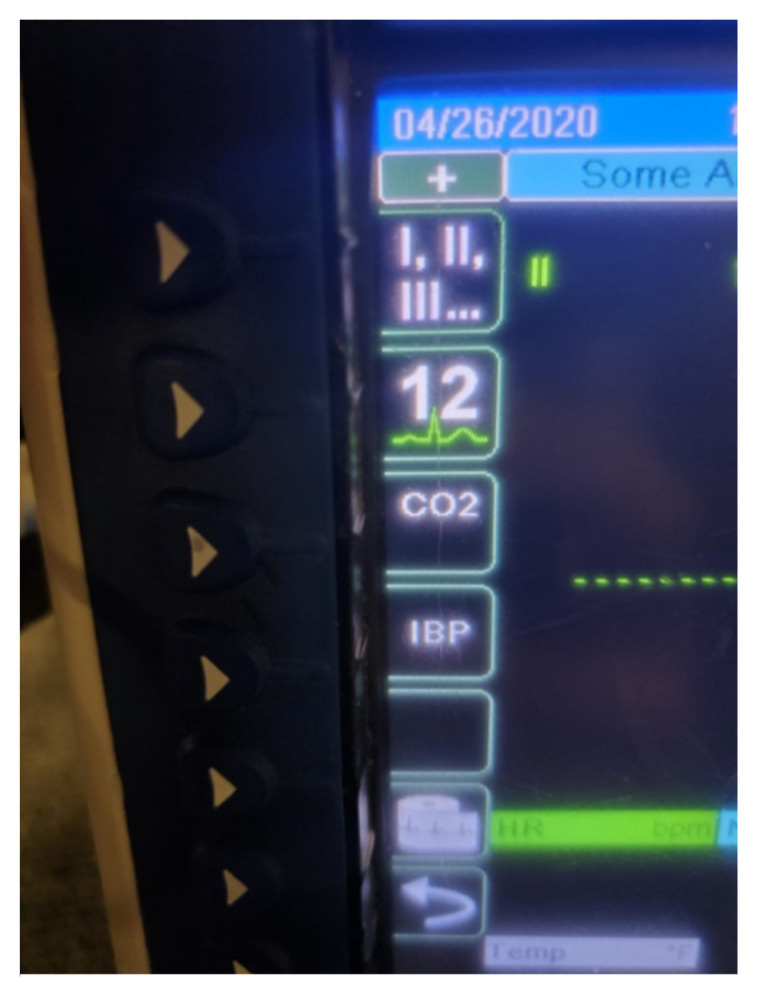
View of monitor depicting "CO2" button on monitor which needs to be pressed to obtain EtCO2 reading Image source: author’s own image

**Figure 4:**
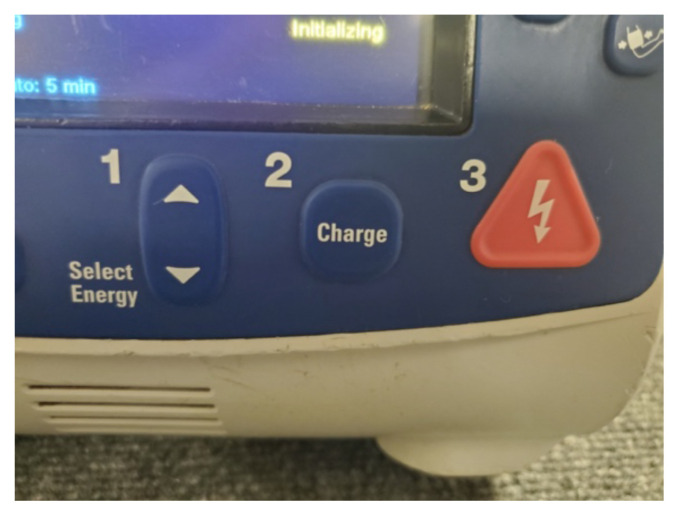
Monitor button placement for defibrillation with associated "1,2,3" numbering associated with order of use. Image source: author’s own image

**Figure 5:**
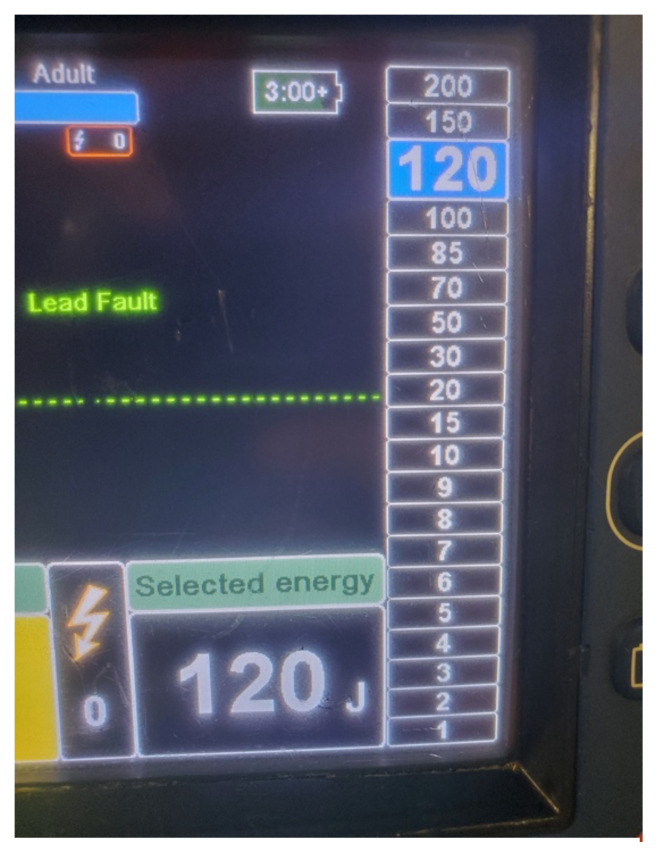
Energy selection on monitor for defibrillation or cardioversion Image source: author’s own image

**Figure 6:**
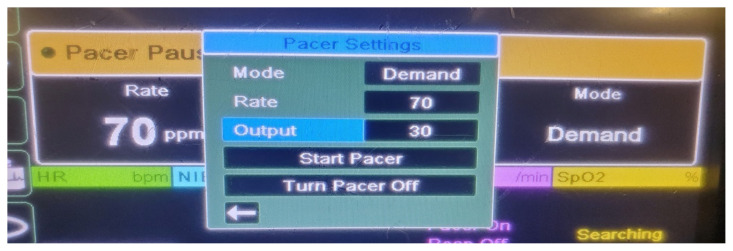
Monitor screen depicting pacer settings Image source: Author’s own image

**Figure 7 f7-jetem-5-3-c82:**
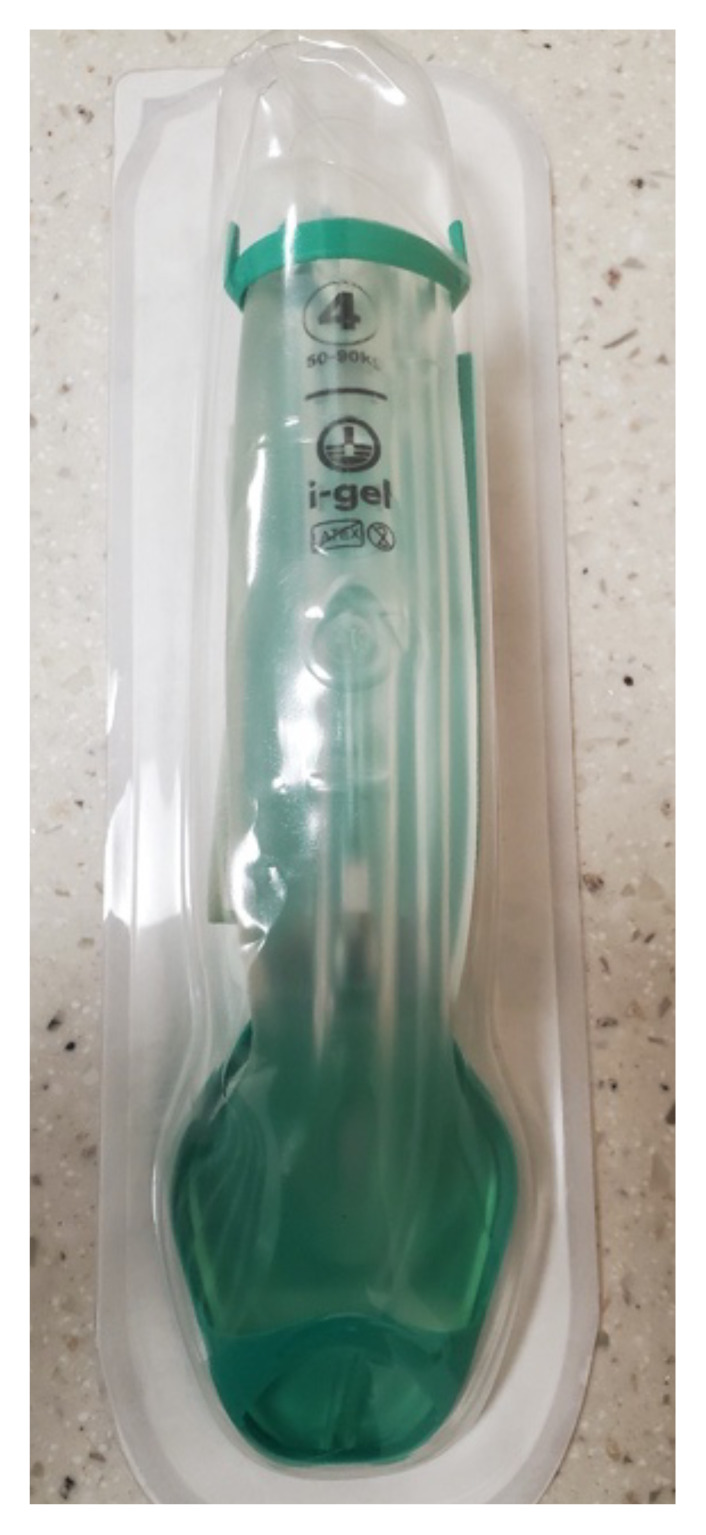
Example of an i-gel size 4 Image source: author’s own image

**Figure 8 f8-jetem-5-3-c82:**
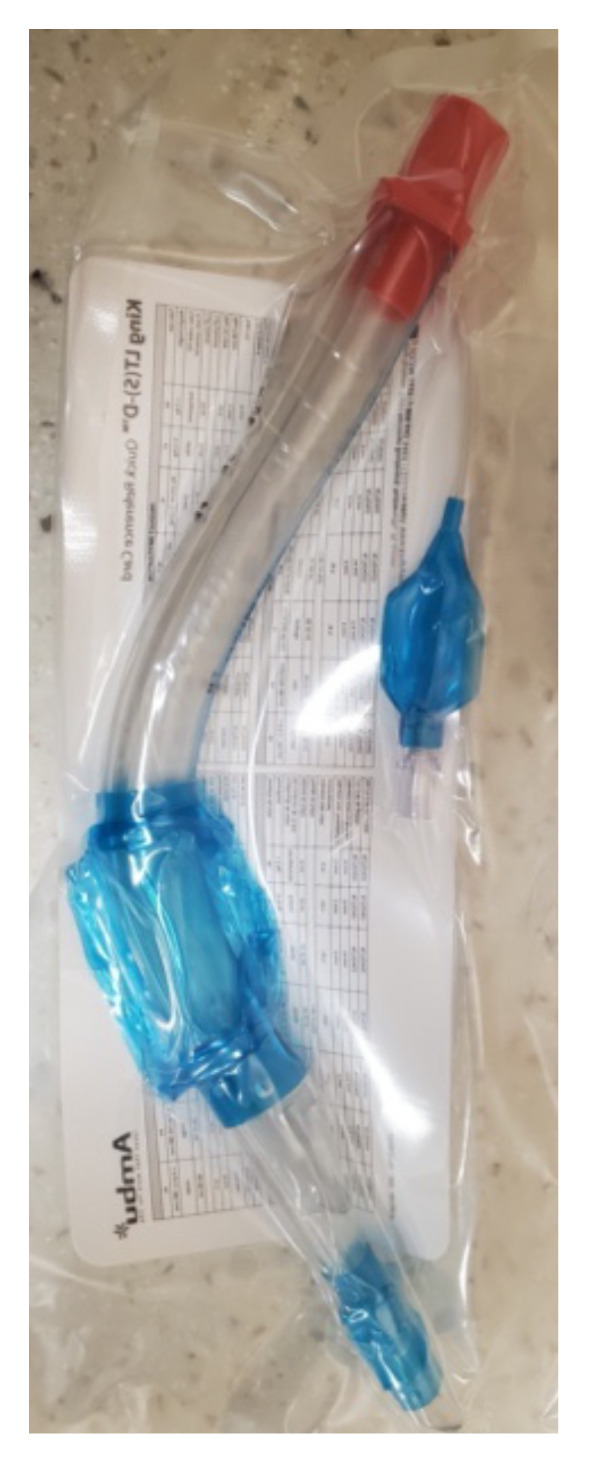
Size 4 King LT Source: Author’s own image

**Figure 9 f9-jetem-5-3-c82:**
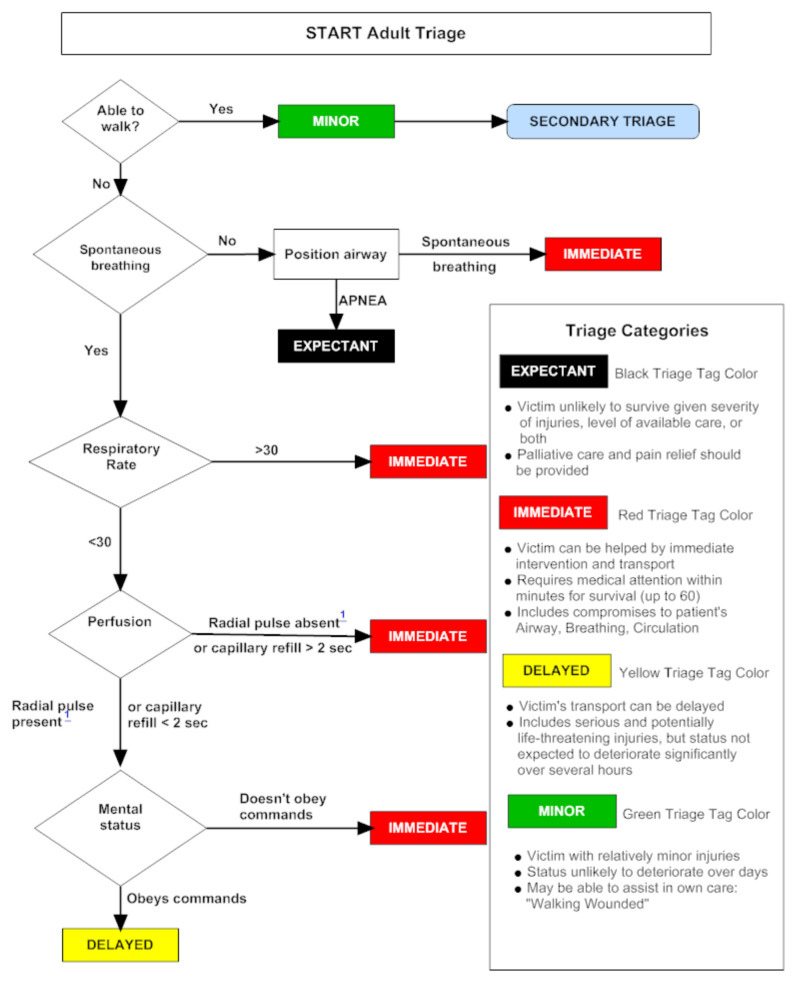
START Triage Algorithm Source: U.S. National Library of Medicine. Available at: https://chemm.nlm.nih.gov/startadult.htm

## DIDACTICS AND HANDS-ON CURRICULUM

### Curriculum Chart

[Table t2-jetem-5-3-c82]

## Appendix A HEMS Program Framework

### Mission profile

The types of flight missions HEMS crews undertake are highly variable and dependent upon many factors, including the area of the country in which providers operate, the type of aircraft they operate, and the role the HEMS provider plays within their regional EMS system. Most commonly HEMS crews are tasked with inter-facility transports of critically ill or injured patients. While not all HEMS operators respond directly to accident scenes, many do for a non-majority share of their flights. Finally, some operators utilize HEMS for other missions such as organ procurement and transport, or medical crew insertion with the intention to return to the receiving facility by traditional ground transport. A small number of programs also participate in search and rescue operations. The specific types of missions which a HEMS program undertakes should be discussed on an individual level with each learner while also making them aware that different programs around the country may be more or less capable based on their infrastructure and resources.

### Crew Makeup

There is no standard configuration for HEMS flight crews within the United States. Medical crews are generally made up of some combination of a nurse, physician, respiratory therapist, or paramedic, typically in a team of two. Crew configuration may also be changed for specific flight missions (eg, a neonatal specific crew may include a respiratory therapist generally not present for adult flights). The necessary training for each position varies between each HEMS operator, but typically includes general licensure in the crew members’ respective field plus critical care and trauma certifications if available to them. Physicians typically have completed a residency in Emergency Medicine; however, other providers with critical care training (adult or pediatric critical care, neonatology, anesthesiology, cardiothoracic surgery) do participate on HEMS crews. International crews often utilize anesthetists. We encourage flight crews to discuss their training and backgrounds with learners.

The role of physicians within flight programs can vary greatly between programs. Most flight programs in the United States do not fly with a physician on board as part of their standard crew but having a physician on board may be beneficial because it can allow for a more experienced provider to provide patient care. Physicians may also have greater training and experience with advanced procedures. Furthermore, flight physicians can provide on-site medical direction and provide guidance outside of established protocols when needed. Regardless of their position as a crew member, HEMS programs will have physician involvement at some level to help with crew member medical training, establishing protocols, as well as quality improvement and quality assurance measures. While the goal of this curriculum is to expose learners to the functions of a HEMS operator and the integration of HEMS operations into their respective regional emergency medical services system, we do encourage learners to be informed of how physicians are involved specifically with their participating program, whether as a crew member or medical director.

The final crew member is the pilot. While the medical crew members typically are focused on patient care, the pilot serves as the point person for aviation and crew member safety. The pilot’s duties include making sure the aircraft and the crew are in a suitable state to safely take a flight mission. Pilots will also make decisions regarding weather (with appropriate crew member input as needed) and if missions can proceed safely in the current weather conditions. There are no standard requirements within the United States for HEMS pilots; however, pilots are generally very experienced. Many have military backgrounds, while some have worked in the tourism industry or private sector. Pilots often undergo training specific to the aircraft they are flying and the vendor for which they are employed. We highly encourage learners to spend time with their crew pilots discussing their backgrounds and previous experiences. Discussions with pilots regarding how they research regional weather predictions and make determinations on weather flight missions can be accepted are particularly high yield. Pilots can also be a wealth of information regarding aircraft of all kinds since they typically have flown multiple types of aircraft.

### Commonly Used Aircraft

There is no standard helicopter favored for HEMS missions in the United States. Programs generally pick an aircraft type which is most suitable for their typical mission profile. Similar to automobiles, there are various aircraft manufactures and vendors. However, one of the bigger dichotomies in HEMS aircraft is that of single versus double engine. There is debate in the field regarding the pros and cons of each and that discussion exceeds the scope of this curriculum. However, two- engine aircraft generally can lift larger payloads and still land in controlled conditions with a single engine in the rare event of an engine failure. Learners are encouraged to discuss their program’s aircraft with the flight crew and pilot. Topics of interest may include typical flight speeds, flight ranges and altitudes, fuel carrying capacity, and payload capacity.

Aircraft may also have different capabilities depending on the avionics installed in the craft. Aircraft with more advanced GPS and autopilot systems and appropriately trained pilots may be able to fly under “Instrument Flight Rules” (IFR). During IFR flight, aircraft will fly at altitudes and headings determined either by predetermined flight paths or under the direct guidance of an air traffic controller. This extra guidance allows aircraft to safely fly during weather where visibility at altitude is limited from the cockpit of the aircraft. This contrasts with “Visual Flight Rules” (VFR) which is the standard for most flights, requiring pilots to be able to see and avoid any potential obstacles in their flight path. Again, the crew’s pilot can be a great source of information regarding the capabilities of the avionics in a particular aircraft.

We suggest learners spend as much time as possible with their associated flight crews to help promote integration into the team as well as to discuss team member backgrounds and training, how the HEMS industry has evolved, and the mission capabilities of the HEMS crew. If interested, further information regarding the above topics and more can be found on the website for the Association of Air Medical Services at https://aams.org/member-services/fact-sheet-faqs/.

## Appendix B Functions of a HEMS Program

### Appropriate Utilization of Air Medical Transport

A key topic for learners is discussion when transport by air is appropriate for a patient. While each HEMS provider will have their own practices, general indications for air transport include the need for expedient travel to a center capable of caring for the ill or injured patient or the need for critical care personnel during transport. ST Elevation Myocardial Infarction (STEMI) and stroke are example medical diagnoses for which HEMS may be utilized to transport a patient to a tertiary center because these have time dependent outcomes for interventions. Another appropriate time-dependent use of HEMS is for patients who are at higher risk for decompensation during transport. This may include premature babies requiring transport to a neonatal intensive care center or ill or injured patients who are geographically isolated and face multiple-hour-long ground transport times. We recommend that crew members discuss their approaches with learners, and that learners review their program’s protocols regarding time-sensitive diagnoses since these will often include time-saving measures for patient care and may differ from standard procedure.

Air transport is also indicated when critical care personnel are needed to safely transport a patient. Examples of this include intensive care patients who require careful ventilator and pharmacological management. Some HEMS programs are also capable of transporting specific patient populations, such as those on extracorporeal membrane oxygenation (ECMO) or intra-aortic balloon pumps. Typically, these crews have specific training in management of the device they are transporting, and often there are not ground services which would accept these patients for transport. We recommend that learners are made aware of the existence of these specialized flight missions, especially if their rotation is at a location which does not participate in these types of missions.

The intersection of the need for critical care personnel and time-dependent care is that of scene flights for traumatically injured patients. While this accounts for a relative minority of HEMS flights, it is also likely the most publicly known use of HEMS since these events often garner news coverage. The maneuverability of small aircraft allows for pilots to insert crew into accident scenes directly, often by landing on roadways or open fields. HEMS crews bring not only critical care expertise, but also, generally, equipment for advanced procedures and resuscitation. This can be especially useful in areas where first responders hold only a basic level of training and may not even be qualified to start intravenous lines. The ability to perform procedures like intubation and chest decompression as well as to perform resuscitation with medications and blood products can make the difference between life and death in the care of pre-hospital traumatic injuries. Once appropriately stabilized, the speed of air travel then decreases the time it takes to reach a capable trauma center. Learners should be given instruction on what equipment and medicines are carried by their program’s crew.

### Integration of Ground-Based and Helicopter EMS into a Unified System

Ground-based and helicopter EMS work in tandem within a region’s EMS system to provide for a common goal of safely and rapidly transporting patients to appropriate care centers. In their function as an inter-facility transport service, HEMS aircraft function very similarly to how a ground-based EMS provider does. They are summoned at the request of a transferring facility, typically for one of the reasons described in the above section. Learners may already have an understanding of this function based upon their prior training as Emergency Medicine residents and experiences with ground-based EMS.

During scene flights, HEMS crews will typically intercept with a ground crew. However, the determination of when to activate an HEMS aircraft for a scene flight can differ between different county and state protocols. Some areas allow for law enforcement officers to activate a HEMS crew without having any other medically trained personnel yet on scene. In this scenario, the HEMS crew would be making primary patient contact. In all other scenarios, the HEMS crew would be assuming care from the local EMS provider. While it is our hope that learners would learn about each of the above scenarios from in-person experience, that may not be possible based on the random nature of flight missions. If near the end of the rotation learners have not participated in a flight of that kind, it would be beneficial to discuss with them typical practices for that interaction. We also support having discussion with learners regarding local and regional EMS protocols regarding how HEMS crews are activated.

Some debate exists of how to best utilize resources when determining if a patient should be transported by ground or air. Air ambulances can generally provide a higher level of care to a patient and transport them to their destination more rapidly. However, situations exist where if the drive time to a receiving facility is fairly short, a ground-based transport which is already on scene may be able to transport a patient to a receiving facility in a shorter period of time than it would take to activate a HEMS crew and subsequently have the patient transported by air. Programs will have individualized knowledge of their flight regions and referral patterns, and we encourage them to discuss these patterns and the reasons behind them with learners.

### Risks and Benefits of HEMS

Similar to ground-based EMS, helicopter EMS is not without some inherent danger. The most feared danger is that of a crash. Accidents are luckily rare in the HEMS industry but can have fatal consequences. In general, transport by air ambulance is safer than many forms of recreational transportation (eg, traveling on a motorcycle or flying a private plane); however, accident rates are roughly four times that of the incredibly safe commercial airline industry. There has been a renewed focus on safety in the HEMS industry since a peak in accidents and fatalities in 2008, and with this a general decline in both accident and fatality rates. Prior to starting a rotation, learners should be made aware that there is a small, but real risk of harm from participation as a member of a HEMS crew.

HEMS work can also put crew members at risk for occupational injuries. Hearing loss from exposure to engine noise is a hazard, as are ergonomic injuries from typically cramped work environments and the physical nature of the work. Occupation hazards can also arise in the form of dangers present at accident scenes (sharp pieces of metal, hazardous material, fire). Learners should be outfitted with all safety equipment provided to every member of the flight crew and given proper instruction on ergonomically safe equipment usage during the teaching time suggested for individual pieces of equipment.

The most clearly proven benefit of HEMS is decreased travel time to a receiving hospital. As described above, time-sensitive diagnoses can benefit from this decreased time to definitive intervention. HEMS transport doesn’t lend itself to double-blinded, randomized controlled trials, however, so the extent of benefit for other flight missions is unclear. Because there is so much variation in regional resources and hospital systems each HEMS provider must determine what missions and in what travel radius that benefits outweigh the risks of flight. We encourage learners to discuss how their program tracks safety data and also discuss national safety trends with their pilots.

## Appendix C Safety in HEMS Aviation

Crew member safety is the highest priority for all HEMS programs. Learners need to have proper instruction regarding how to safely operate in and around their program’s aircraft. We encourage programs implementing this curriculum to provide learners with the same instruction that they provide newly employed flight crew members prior to allowing them to fly. To comply with Federal Aviation Association (FAA) requirements, learners will either need to complete Air Medical Resource Management (AMRM) training prior to the rotation or complete a pre-flight briefing prior to each flight. Our program also utilizes safety training videos provided by our aircraft operations company, Metro Aviation. We encourage learners to be given access to safety training documents and videos specific to the aircraft and operator whom they will be riding along with. If no such media exists, learners can visit https://youtu.be/6YCN1WAL4OY to view the publicly published video Metro Aviation publishes.

In addition to the operator-provided safety media, we suggest learners are paired with a crew pilot to review the below topics. All HEMS pilots will be able to teach these topics (as pilots need to have understanding of the topics to be compliant when giving FAA required crew briefings), and so the information below is left intentionally vague. The below list is not meant to be all inclusive but is provided so that learners may be provided a copy for their own review and identify any shortcomings in their education, since certain topics may need to be reviewed before learners feel they can safely participate in flights.

1. Proper safety equipment
  - Learners should be provided with properly fitting, flame retardant flight suits and flight helmets.
  - Fit should be checked to ensure equipment will function as intended.
  - Learners should also wear appropriate footwear. We suggest leather boots, and some programs may encourage use of steel-toed footwear.
2. Safety in the aircraft
  - Learners need to properly demonstrate use of the seatbelts and how to brace themselves in a crash position.
  - Learners should also know locations of and how to use the fire extinguishers within the aircraft.
  - Learners need to be able to identify emergency exits and demonstrate knowledge of how they operate.
  - Learners who will be riding in the cockpit of the aircraft need to be able to demonstrate the following:
    - Methods for engine shutdown in event of a crash
    - Activation of the emergency location transponder
    - Oxygen shutoff
    - Usage of rotor brake
    - Usage of emergency procedures checklist for pilot aid
3. Safety around the aircraft
  - Learners should be given instruction on how to properly perform a pre-flight “walkaround” while visually inspecting the aircraft.
  - Learners need to understand how to safely enter and exit an aircraft when the main rotor is turning.
    - This does not apply to programs which do not “hot load.”
    - Methods of nonverbal communication between the pilot and learner which signals that the learner may safely approach the aircraft while it is running should be reviewed.
  - Learners need to demonstrate safe practices around the tail rotor of the aircraft.
    - Most learners should be instructed to never enter the space behind the vertical stabilizer on the tail of the aircraft.
4. Safe flight practices
  - Learners should be given instruction on how to properly call out potential dangers to their pilot by using the clock-face system and position relative to the horizon.
    - For example, an aircraft flying below and to the left side of a helicopter should be described to a pilot as “aircraft nine o’clock, low.”
  - Learners should be instructed on proper communications during flight.
    - This includes limiting all non-aviation and non-medical discussion during all phases of flight.
    - This also includes the time of “sterile cockpit” during critical phases of flight (ie, take-off and landing) during which there should be no talking except to aid in aviation.
  - Learners should be instructed on the physiologic changes of flight.
    - While this is unlikely to be significant at the altitudes which most medical helicopters fly, ongoing sinus congestion can cause significant pain.
    - The effects of lack of sleep, alcohol, caffeine, and other medicines can be altered or increased, and learners should be made aware of this.

## Appendix D Flight Resident Physician Shift Expectations

Prior to the start of their rotation, learners are provided the below document detailing expectations for them while on shift. This document is provided as an example so programs can create their own shift expectation documents. Suggested topics include the following:

1. Expected arrival time for shift
2. Start times of any meetings or briefings which learners are expected to attend
3. Crew member duties need to be ready to accept a flight
4. Expectations of a learner while not on a flight mission

### Flight Resident Physician Shift Expectations

- Arrive several minutes early to change in to your flight suit and be prepared to assist in aircraft and equipment checks at the start of your shift.
- Attend all briefings that occur during your shift:
  - Briefings are at: 0735, 0945, 1935, 2145.
- Perform the equipment and aircraft checks with the crew, as well as assist in all cleaning and other inventory.
- Obtain your helmet and life-jacket for the shift.
  - Make sure to remove your helmet from the helicopter at the conclusion of your shift and replace it in the inventory locker.
- Be upstairs to greet and assist all incoming aircraft.
  - For our program’s aircraft – either accompany the crew as they drop off the patient or assist in restocking. This is a great opportunity to discuss the case with the crew and learn.
  - For aircraft from other programs – assist in any way possible. If possible, ask their pilot if you can look at their aircraft because many programs use helicopters other than EC-135/145s.
- During your shift, attend all relevant Critical Care Transport meetings:
  - Education Meeting – 1^st^ Monday of every month, 0900, E3/217.
  - Quality Meeting – 1^st^ Monday of every month, 1100, E3/217.
  - Operations Meeting – 1^st^ Monday of every month, 1230, E3/217.
  - Safety Meeting – 3^rd^ Tuesday of every month, 1400, E5/217.
  - MF RN Education – 2^nd^ Tuesday of every month, 1630, E5/714.
  - All CCT Meeting – 2^nd^ Tuesday of odd months, 1800, E5/714.
  - All CCT Education – 2^nd^ Tuesday of odd months, 1900, E5/714.
- On Thursdays, check with the pilot to see if he is comfortable with you attending Residency didactics, and review the best way to ensure your return to the helipad as quickly as possible in the event of a flight.
- If you are not able to go with your crew on a flight for whatever reason, you should still be at the helipad upon their return to review the case and assist the crew.
- If weather is very unfavorable for flying, consider accompanying our ground teams on ground transports if appropriate.
- Use "downtime" to work on residency-related projects or educational materials. There is also a program textbook library available.
- Resident Evaluation Cards - we’ve collaborated with our residency leadership to come up with shift feedback cards specific to your program. The goal is to have these done for every shift, if possible. It is your responsibility to bring these to the flight crew to be filled out, and then they should be placed in the metal black return box. These cards are colored bright pink and are located in the second call room.
- Topic of the shift - As you may have noticed, the evaluation card has an area at the bottom asking if the “Topic of the shift” was reviewed. We have come up with a selection of topics to be reviewed by you with the flight crew during every shift. This will not only prove educational but will also serve as a starting point for discussion and interaction between the on-duty crew and yourself. Since there are 12 shifts during the elective month, we have come up with 12 topics to be covered. These will be laminated and placed in the resident’s call room as well, and it will again be your responsibility to review them and discuss them with the staff. See this as a starting point to broach different subjects, and it will likely be easiest to do this during the equipment checks at the start of your shift.
- Patches – these are to be worn on your flight suit. They are kept in the Communications Center.
  - Observer patch should be worn when in the Observer phase. Your primary focus during this phase is on helicopter safety and beginning to understand transport medicine.
  - Resident Flight Physician patch should be worn during the Solo-In-Training phase and the Solo phase, until you get your personalized patch.

## Appendix E Radio Usage

Each program and aircraft will have their own specific radio configuration. Below we include an example of the “guide to radio usage” we provide learners, which has photographic aids to help in explanation. Programs should create similar documents to enable learners to properly communicate during flight and with the HEMS base or ground personnel. We recommend including the following suggested topics:

1. Description of various radio banks present in the aircraft
  - For example, our aircraft contains 4 radios, of which only one channel is commonly used by the med crew for communications; the rest are primarily for pilot usage.
2. Description of various channels and their uses for the medical crew
  - We have designated channels for communications with our base, our county communications center, various ER channels, and local channels for contacting ground EMS or fire crews.
  - Learners need to be shown how to change through the various channels to reach their desired target for communication.
3. Description of how to utilize the intercom system (ICS) within the aircraft and how to trouble shoot common problems with the ICS

### Guide to Radio Usage

#### EC-145

- VHF1 and VHF2 are ATC/Aviation channels.
- FM1 is the VHF/UHF channel, which includes MARC II, EMS B, EMS C, Med 8, and Med 10.
- IRIS is for talking to Metro Operational Command Center or to Med Base via satellite.
- When the buttons are out – you can hear that channel.
  ○ The only exception is if you have that channel selected – then you can hear it regardless.
- The selector knob determines which channel you will talk on – for medical personnel this will always be 3.
- On FM1 (3) – you change the channel using the 4 (MUP) and 7 (MDN) channels.
  ○ If you get lost – hit the HOME button and it will default to MED 10.
  ○ Please do not adjust the black rotary dial on the radio control head.
- In the copilot (front) seat – hold the PTT toggle down to transmit out on the radio.
- The Vox knob controls your Vox sensitivity.
- The IC VOL knobs control the volume of ICS as well as main radio.
- In back – everything is the same, except you use the button on your Carter box to transmit out.
- In general – you should assume that the radios are set up correctly, and pause and reassess so that when you make adjustments these can be made discretely and intentionally.[Fig f10-jetem-5-3-c82][Fig f11-jetem-5-3-c82]

#### EC-135

- COM1 and COM2 are ATC/Aviation channels.
- The VHF/UHF channel includes MARC II, EMS B, EMS C, Med 8, and Med 10.
- IRIS is for talking to Metro OCC or to Med Base via satellite.
- When the buttons are out – you can hear that channel.
  ○ The only exception is if you have that channel selected – then you can hear it regardless.
- Pushing the small button underneath the channel will select it and turn the light on – this is the channel you will transmit on.
- On VHF/UHF – you change the channel using the 4 (MUP) and 7 (MDN) channels.
  ○ If you get lost – hit the HOME button and it will default to MED 10.
  ○ Please do not adjust the black rotary dial on the radio control head.
- In the copilot (front) seat – hold the ICS/TX toggle down to transmit out on the radio.
- The Vox knob controls your Vox sensitivity.
- The ICS knob controls your ICS volume, and MVOL controls the overall volume.
- In back – everything is the same, except you use the button on your Carter box to transmit out.
- In general – you should assume that the radios are set up correctly, and pause and reassess so that when you make adjustments these can be made discretely and intentionally.[Fig f12-jetem-5-3-c82][Fig f13-jetem-5-3-c82]

## Appendix F Advanced Topics in Critical Care Transport

The below worksheets serve as example discussion topics for learners and can be used to ensure that uniform instruction is given across multiple learners. The below pages contain some information specific to the authors’ program, and these topics should be edited or changed to be pertinent to the equipment and practices in place at the program wishing to implement this curriculum.

1. Tranexamic Acid and Blood Product Administration
2. DOPES Protocol
3. Suction Assisted Laryngoscopy and Airway Decontamination Technique for Intubation
4. Use of the Bougie
5. High Peak Airway Pressure Alarms
6. Ventilation in the Difficult-to-oxygenate Patient
7. Rapid Sequence Intubation Medications and Post-Intubation Sedation and Analgesia
8. Use of the Monitor for End-tidal CO2, Pacing, and Defibrillation
9. Management of Elevated Intracranial Pressure and the use of hyperosmolar agents
10. Failed Airway Procedures –Cricothyrotomy
11. Extra-glottic Devices
12. Pelvic Binders

### Learning Objectives

1. Learners should know indications and dosages for blood products and tranexamic acid administration.
2. Learners should be able to describe how to trouble shoot a scenario in which a ventilated patient unexpectedly becomes hypoxic.
3. Learners should demonstrate understanding of how any advanced procedures (suction assisted laryngoscopy, bougie use, cricothyrotomy, extra-glottic device placement, pelvic binder placement) are performed.
  - Learners should only participate in medical care to the level of crew member comfort and the learner’s level of training, so demonstration of proficiency with the procedure is not required.
4. Learners should demonstrate approaches to ventilation in patients who are difficult to oxygenate or trigger peak airway pressure alarms on the ventilator.
5. Learners should know all commonly used medicines and appropriate dosages for medications used for rapid sequence intubation and post-intubation sedation.
6. Learners should understand how to use various functions on a cardiac monitor as well as end-tidal CO2 monitoring.
7. Learners should know indications and dosages for hyperosmotic agents to be used in situations of elevated intracranial pressure.

#### 1. Tranexamic Acid and Blood Product Administration

- Blood products carried:
  ○ Packed Red Blood Cells (PRBCs)
  ○ Thawed Plasma
  ○ Tranexamic Acid (TXA)
- Administration should be considered for patients:
  ○ In hemorrhagic shock from recent trauma.
  ○ Hemorrhagic shock from other bleeding, such as ruptured abdominal aortic aneurysm (AAA) or gastrointestinal (GI) bleeding.
  ○ Coagulopathic patients, whether from intrinsic disease or anticoagulant medications.
- Trauma Patients:
  ○ Consider blood products in any patient with evidence of significant blood loss or deterioration consistent with hemorrhagic shock or early coagulopathy.
  ○ Hang plasma and PRBCs simultaneously using the Y-site blood administration set.
  ○ Plasma can potentially be used to prime the tubing and should be given first.
  ○ Immediately follow with PRBC.
  ○ Ideally utilize both pressure bags as well as fluid warmer.
  ○ TXA:
    ▪ Indicated in adult patients who suffered traumatic injuries within the last 3 hours.
    ▪ 1 g of TXA should be given over 10 minutes as a slow push or infusion.
- Nontraumatic bleeding:
  ○ Prioritize PRBC infusion, although plasma is likely to be useful adjunct.
  ○ Consider TXA if known onset within 3 hours.
  ○ Ideally acquire blood products from referring facility in addition to our own supply.
  ○ Follow principles of hemostatic resuscitation and allow permissive hypotension to minimize crystalloid usage.
- Coagulopathic patients:
  ○ Prioritize plasma infusion, although PRBC may be helpful to replete the acute blood loss.
  ○ Acquire blood products from referring facility, including platelets if possible/indicated.
  ○ Depending on reason for coagulopathy, inquire at referring facility about availability of other adjuncts such as DDAVP, Factor VIII, and prothrombin complex concentrate (PCC).

#### 2. DOPES Protocol

- Mnemonic to be used in intubated patients with unexpected hypoxia or deterioration.
- D = Displacement:
  ○ Check the placement of the endotracheal tube.
    ▪ The tube may have entered the right mainstem or may have withdrawn out of the trachea.
  ○ Check the depth of the endotracheal tube and compare to initial depth.
    ▪ All endotracheal tube measurements should be taken in reference to the patient’s incisors, and not the gums.
  ○ If unable to auscultate (due to constraints during flight), consider using ultrasound to assess for bilateral lung sliding to rule-out mainstem placement.
- O = Obstruction:
  ○ Check for obstruction of the endotracheal tube as well as the ventilator circuit.
  ○ Consider deep suctioning of endotracheal tube.
  ○ Evaluate for kinking of the ventilator circuit tubing as well as connections to the ventilator.
- P = Pneumothorax:
  ○ Assess for higher peak inspiratory pressures.
  ○ Assess for equal chest rise.
  ○ If unable to auscultate, consider using ultrasound to assess for pneumothorax.
  ○ If high suspicion, have low threshold to perform needle decompression or finger thoracostomy.
- E = Equipment:
  ○ Evaluate for equipment failure, specifically the ventilator.
  ○ Check all connections and alarms.
  ○ Check EtCO2 waveform and pulse oximetry.
  ○ Ensure adequate oxygen supply.
  ○ Ensure patency of pilot balloon.
  ○ If unable to quickly fix issue, remove the patient from the ventilator and bag until stable and can reevaluate.
- S = Stacking:
  ○ Evaluate for breath stacking:
    ▪ Compare expired volume to inspired volume.
    ▪ Evaluate for chest wall rise, tympany.
    ▪ Evaluate for AutoPEEP (auto-positive end expiratory pressure) – can use expiratory hold to assess for this.
  ○ If high suspicion – disconnect and compress chest and allow patient to fully exhale.

#### 3. Suction assisted laryngoscopy airway decontamination (SALAD) Intubation

Suction assisted laryngoscopy airway decontamination (SALAD) is an intubation technique used to prevent airway soiling in a patient with profuse emesis.

- Technique:
  1. Decontaminate the oropharynx with your suction prior to inserting the laryngoscope blade.
  2. Lead the blade with the suction while hugging the anterior surface of the tongue to avoid soiling the illumination/optics with emesis.
  3. As the blade moves into place, continue to decontaminate the hypopharynx and then place the suction into the proximal esophagus.
  4. With the suction still in the esophagus, reposition it to the left corner of the mouth. This can be facilitated by a slight withdrawal and reinsertion of laryngoscope blade. The suction should now be pinned to the left corner of the mouth.
  5. You may need to slightly rotate the blade leftward to facilitate passing of the endotracheal tube into the mouth and through the cords.
  6. Inflate the cuff and suction the tracheal tube and trachea prior to ventilation to avoid any further aspiration.

#### 4. Using the Bougie as an airway adjunct

- Uses of a bougie:
  ○ Tracheal intubation during laryngoscopy, especially in difficult airways.
    ▪ Best used with standard geometry laryngoscopy blades, whether Direct or Video.
  ○ Tracheal intubation with supraglottic airway device in place.
  ○ Endotracheal tube exchange.
  ○ Cricothyrotomy – scalpel, finger, bougie technique.
- Bougie assisted endotracheal intubation:
  ○ Typically held 20–30 cm proximal to the coudé tip.
  ○ Ideally held in a “shaka” grip (Figure 1), with fingers above and below the bougie and your thumb posterior to the bougie.
  ○ Insert via side of the mouth; rotation allows for control of the bougie in the vertical plane.
  ○ Ideally you will visualize the bougie pass into the trachea. This should be supplemented by a tactile “click” as it passes along the tracheal rings.
  ○ At about 30–40 cm depth, there should be a hold up at the carina. If this does not occur, you are likely in the esophagus.
  ○ Once the bougie is in the trachea, keep the laryngoscope blade in the mouth and railroad the endotracheal tube over the bougie while maintaining your view and visualizing the tube pass through the cords.
  ○ The ETT can railroaded by an assistant or can be preloaded onto the bougie.

#### 5. High Peak Airway Pressure (PAP) alarms

- Work of breathing is measured in terms of pressure which is separated into two components:
  ○ Airway resistance
  ○ Lung compliance
- Elevations in airway pressure causing high PAP alarms are either due to increases in airway resistance or decreases in lung compliance.
- Peak pressure – total amount of airway pressure delivered to overcome resistive and elastic work of lung.
- Plateau pressure – airway pressure at the end of inspiration when flow through airway has finished (resistive work is zero). Measured by performing an inspiratory pause – only do this is in properly sedated patients.
- High peak pressures (and normal plateau pressures):
  ○ Causes include obstructive airway disease, narrowed ETT (mucous plug), tube kinking, bronchospasm, turbulent air flow.
  ○ Not overly concerning, should not cause alveoli damage.
  ○ May want to increase peak pressure limit on ventilator so can deliver full tidal volume.
  ○ Treatment - bronchodilators or remove mechanical obstruction.
- High plateau pressure – measured by inspiratory pause
  ○ Pneumothorax, acute respiratory distress syndrome (ARDS), pulmonary edema, pneumonia, right mainstem intubation, breath stacking
  ○ Increases with increasing tidal volume (TV), poor lung compliance, and positive end expiratory pressure (PEEP)
  ○ In ARDS, increase PEEP and decrease TV to maintain plateau pressure < 30 cm H20
  ○ In obese patients – sitting them up can significantly reduce airway pressures
- Breath stacking (auto PEEP) - measured by expiratory pause.
  ○ Treat by:
    ▪ Maximizing expiratory time by adjusting inspiratory to expiratory (I:E) ratio.
    ▪ Decrease respiratory rate.
      ▪ Disconnect ventilator and manually push down on chest to force exhalation.

#### 6. Considerations in Difficult-To-Oxygenate Patients

- Improving oxygenation is achieved by adjusting two main factors:
  ○ Increasing the fraction of inspired oxygen (FiO2).
  ○ Increasing airway pressure by increasing PEEP.
    ▪ Recruitment of additional lung surface area for diffusion.
    ▪ Effect is not immediate (up-titrate 2 cm H20 every 15–30 minutes).
    ▪ Can have negative hemodynamic effects from increased intrathoracic pressure.
- Unilateral lung disease (ex: pneumonia, hemothorax, pleural effusion).
  ○ Low TV.
  ○ Lateral decubitus position with good lung down – increases blood flow to good lung.
- Bilateral lung disease (ex: ARDS or cardiogenic pulmonary edema).
  ○ Low TV and higher PEEP.
  ○ Consider paralysis to improve ventilator compliance.
  ○ Proning – but difficult to achieve in transport.
- Goal oxygen saturations of 94%–96% in most patients.
- Lower saturations of 88%–95% are tolerated with ARDS.
- Most patients can also tolerate hypercapnia better than hypoxia – especially if no other metabolic abnormalities such as concurrent metabolic acidosis.
- Elevate head of bed to at least 30 degrees.
- Consider recruitment maneuvers.
- Can use ARDSNet table (Table 1) to titrate FiO2 and PEEP.
  Do not be afraid to increase PEEP to 10–20 mm Hg if necessary and safe for the patient – closely monitor for complications.

**Table 1:** Suggested PEEP and FiO2 settings for ARDS

Lower PEEP/Higher FiO2								
**FiO2**	**0.3**	**0.4**	**0.4**	**0.5**	**0.5**	**0.6**	**0.7**	**0.7**
**PEEP**	**5**	**5**	**8**	**8**	**10**	**10**	**10**	**12**

**FiO2**	**0.7**	**0.8**	**0.9**	**0.9**	**0.9**	**1.0**
**PEEP**	**14**	**14**	**14**	**16**	**18**	**18–24**

Higher PEEP/Lower FiO2								
**FiO2**	**0.3**	**0.3**	**0.3**	**0.3**	**0.3**	**0.4**	**0.4**	**0.5**
**PEEP**	**5**	**8**	**10**	**12**	**14**	**14**	**16**	**16**

**FiO2**	**0.5**	**0.5–0.8**	**0.8**	**0.9**	**1.0**	**1.0**
**PEEP**	**18**	**20**	**22**	**22**	**22**	**24**

#### 7. Rapid Sequence Intubation (RSI) and Post-Intubation Sedation and Analgesia

##### Induction/sedation agents

- Ketamine
  - Dose: 1–2 mg/kg IV or 4 mg/kg IM (induction), 0.25–0.5 mg/kg bolus or 1 mg/kg/hr infusion (post intubation sedation).
  - Onset: 60–90 sec.
  - Duration: 10–20 mins.
  - Good for hemodynamically unstable patients and reactive airway disease (causes bronchodilation), preserves respiratory drive (awake look/intubation or delayed sequence intubation), provides some analgesia; use caution in cardiovascular disease (causes hypertension/tachycardia), DOES NOT increase intracranial pressure.
- Etomidate
  - Dose: 0.3 mg/kg (induction).
  - Onset: 10–15 seconds.
  - Also safer for hemodynamically unstable patients, but can cause adrenal suppression.
- Propofol
  - Dose: 1–2 mg/kg (induction), 0.2–2 mg/kg bolus and 5–50 mcg/kg/min (post intubation sedation).
  - Onset: 12–45 secs.
  - Duration: 5–10 mins.
  - Generally not used for RSI; useful in status epilepticus, short onset/duration, good post intubation sedation in hemodynamically stable patients.
- Midazolam
  - Dose: 1–5 mg bolus, 2–10 mg/hr infusion.
  - Onset: 60–90 secs.
  - Adverse effect: myocardial depression can result in hypotension.
  - Not recommended for RSI; good for post intubation sedation in patients with less favorable hemodynamics but can contribute to delirium.
- Fentanyl
  - Dose: 50–100 mcg bolus, 50–200 mcg/hr infusion.
  - Onset: < 60 secs.
  - Duration: dose dependent, ~30 mins for 1–2 mcg/kg.
  - Generally used for post intubation analgesia; can cause hypotension but most hemodynamically neutral IV analgesia.

##### Paralytic agents

- Succinylcholine
  - Dose: 1.5 mg/kg.
  - Onset: 45–60 seconds (looks for fasciculations).
  - Duration: 6–10 mins.
  - Contraindicated in hyperkalemia, >5 days post burn/crush spinal injury, neuromuscular disease. Preferred for short duration so can reassess neuro status.
- Rocuronium
  - Dose: 1.2 mg/kg.
  - Onset: 60 secs.
  - Duration: 30–90 mins.
  - Long duration can be drawback if want to evaluate neuro status; sugammadex for reversal. Ensure adequate post intubation sedation and analgesia during prolonged period of paralysis.

##### Pearls

- Consider increased paralytic dose and decreased sedative dose in shock state patient.
- Ketamine and etomidate in high enough doses can still cause hypotension.
- Always – resuscitate before intubation.

#### 8. Monitor Use for End Tidal CO2, Defibrillation, Cardioversion, and Pacing

*Author’s note: this section is specific to the brand of monitor used at our program and needs to be adjusted based on the equipment in use at an implementing program*.

##### End Tidal CO2 Monitoring

- End Tidal CO2 (ETCO2) Monitoring Indications:
  ○ Any intubated patient to ensure proper ventilation.
  ○ Any patient where real time monitoring of adequate ventilation metrics is necessary for ideal care.
- Description of use:
  ○ In un-intubated patients – utilize the nasal cannula EtCO2 device.
  ○ In intubated patients – utilize the in-line EtCO2 device.
  ○ The device will plug in on the left side of the Zoll monitor (Figure 2) – then ensure that the CO2 button on the front of the monitor is selected in order to show the tracing on your monitor screen (Figure 3).

##### Defibrillation

- Defibrillation Indications:
  ○ Ventricular tachycardia without pulse.
  ○ Ventricular fibrillation.
  ○ Any unstable rhythm without identified QRS rhythm.
- Process of defibrillation:
  ○ Place Pads on patient – either anterior and lateral or anterior and posterior.
  ○ Select “Energy Level” (Figure 4).
    ▪ Adults: 120–200 Joules (Figure 5).
    ▪ Pediatrics: Start at 2 J/Kg, followed by 4 J/Kg.
  ○ Press Charge Button on monitor.
  ○ Wait for audible indication that defibrillator is charged.
  ○ Verbalize “CLEAR” and visually ensure patient is clear.
  ○ Press “SHOCK” on ZOLL monitor.
- Safety
  ○ If the electrical energy is not discharged properly, the energy could cause personal injury or death to the operator or bystander.
  ○ If you wish to cancel the defibrillation, press the “Disarm” softkey.
  ○ The monitor will automatically cancel the charge after 60 seconds.

##### Synchronized Cardioversion

- Indicated for any unstable rhythm where a QRS can be identified.
- Start by placing pads on patient:
  ○ Either anterior and lateral or anterior and posterior.
- Press “SYNC” button:
  ○ Visually ensure that **QRS Marker** on monitor is present.
  ○ Patient must have an R-wave in order to sync.
- Select “Energy Level”:
  ○ Adults: 120 – 200 Joules.
  ○ Pediatrics: 0.5–1 J/kg can increase to 2 J/Kg, if needed.
- Press “Charge” Button on monitor:
  ○ Wait for audible indication that monitor is charged.
- Verbalize “CLEAR” and visually ensure patient is clear.
- Press “SHOCK” on monitor.

##### Pacing

- Indicated for symptomatic and/or hemodynamically unstable bradycardia.
- Place pads on patient – either anterior and lateral or anterior and posterior.
- Press “Pacer” Button on monitor to display the pacer settings menu (Figure 6).
- Set “Mode”:
  ○ “Demand” should be selected by default.
  ○ Use up and down arrow key on right to navigate this window.
- Select “Rate”:
  ○ typically, 70–80 bpm but can be adjusted based on patient perfusion.
- Ensure everyone is clear of patient and verbalize “Starting Pacer.”
- Select “Output” in milliamps:
  ○ Slowly increase the milliamps until capture is obtained.
  ○ Adjust output to 5–10 milliamps above that level to ensure continued capture.
- Select “Start Pacer”:
  ○ Monitor patient heart rate and pulse to confirm capture.
  ○ Electrical capture can be determined by the presence of a widened QRS complex, the loss of intrinsic rhythm, and the appearance of an extended and sometimes enlarged T-wave.
  ○ Mechanical Capture is determined by feeling a pulse at the set rate.

#### 9. Management of Elevated Intracranial Pressure (ICP) with hyperosmotic agents

##### Overview

In the patient with severe closed traumatic head injury or non-traumatic injury demonstrating severe elevation in ICP, the use of hyperosmotic agents can provide temporizing reduction of ICP until definitive neurosurgical intervention can be provided. HEMS is often called for emergent transport of these patients to reach neurosurgical service.

##### Approach to head injured patients

- Acutely brain injured patients have outcomes tied to their cerebral perfusion pressures.
- Cerebral perfusion pressure (amount of blood getting to the brain) is determined by the difference in the mean arterial pressure and the ICP.
  ○ ICP can be directly measured via invasive intracranial monitoring; however, this is often not available for HEMS crews.
  ○ Mean arterial pressures can be determined by invasive or non-invasive blood pressure monitoring.
- Clinical symptoms/signs of elevated ICP:
  ○ Unilaterally or bilaterally dilated pupils
  ○ Intractable seizures
  ○ Declining mental status
  ○ Progressing neurologic deficits
  ○ Hemodynamic instability consistent with increased ICP (bradycardia, hypertension)
- Current recommendations do not advocate for prophylactic use of hyperosmotic agents, yet hypertonic saline may offer a benefit prophylactically because it also supports normotension, therefore cerebral prefusion pressure.

##### Use of hyperosmotic agents

- The most commonly used agents are hypertonic saline and mannitol.
- Indicated for patients with a severe traumatic head injury or non-traumatic cause leading to severe elevation of ICP.
- Contraindications:
  ○ In imminent herniation situation there is unlikely to be any absolute contraindications.
  ○ Relative indications would be if serum sodium was known to be already supratherapeutic.
- Mannitol dose is 1 g/kg over 10–20 mins.
- 3% Hypertonic Saline given for imminent herniation or evidence of declining mental status.
  ○ Adult dose is 150–250 ml given over 10–20 mins, may be repeated.
  ○ Pediatric dose is 3–5 ml/kg given over 10–20 mins, may be repeated.

##### Further management of head injured patients

- Elevate head of bed > 45 degrees whenever possible.
  ○ Increased venous drainage via gravity.
  ○ May not be possible when patient is in spine precautions.
- Loosen cervical collar to allow for venous drainage when possible.
- Maintain normotension – Systolic BP >90 and <140–160 mm Hg.
- Maintain Adequate oxygenation SpO2 94–99%.
- Intubate for GCS < 8.
- Utilize RSI agents.
  ○ Ketamine has been proven not to increase ICP.
- Treat pain and provide adequate sedation post-intubation.

#### 10. Cricothyrotomy

- Overview
  ○ Open procedure performed to secure the airway via an incision in the cricothyroid membrane.
  ○ Is a type of emergency surgical airway (ESA).
  ○ Distinct from needle cricothyroidotomy, which is an alternative approach to “front of neck access” procedures to salvage an airway.
- Indicated in “can’t intubate, can’t ventilate” scenarios.
- Contra-indications:
  ○ The ability to secure an airway with less invasive means.
  ○ airway trauma that renders access via the cricothyroid membrane futile.
    ▪ Eg, laryngeal fracture, tracheal transection.
    ▪ In the above cases, tracheostomy should be performed, or access achieved via the traumatic airway opening.
  ○ Children < 10 years of age
    ▪ Young children are prone to laryngeal trauma, and they have a higher incidence of postoperative complications.
    ▪ Performing needle cricothyrotomy is generally advised; however, life-saving surgical cricothyroidotomy has been successfully performed in children.
- General approach to an emergency surgical airway:
  ○ Numerous techniques have been described; our preferred approach is the ‘scalpel-finger-bougie’ approach described below.
  ○ In an anticipated difficult airway requiring emergency intubation, a “double set up” approach should be used if possible.
    ▪ One person attempts intubation.
    ▪ Another person prepares to perform the ESA.
    ▪ Dons PPE.
    ▪ Marks the skin with surgical pen to identify cricothyroid membrane.
    ▪ Locates cricothyroid membrane area and anesthetizes area if possible.
    ▪ Has equipment opened and ready to proceed when necessary.
    ▪ In a true emergency, there may not be time for sterile preparation of the skin.
  ○ Consider sedation (eg, ketamine) – there may not be time in a true emergency, and the patient will become obtunded as hypoxia supervenes.
  ○ ESA is a tactile procedure and must be able to be performed without visual cues.

##### Scalpel-finger-bougie approach

- Equipment:
  ○ scalpel blade (eg, size 10)
  ○ bougie
  ○ size 6-0 endotracheal tube (ETT) or tracheostomy tube
- Performing the Procedure:
  ○ Extend neck in supine position to make anatomy more accessible by palpation
    ▪ Can utilize ultrasound (if time and available).
    ▪ The airway has priority over suspected c-spine injury.
  ○ Stabilize the thyroid cartilage with the non-dominant hand.
  ○ Dominant hand holds scalpel and rests on the patient’s sternum for stability and support.
  ○ Create a 4 cm vertical incision through skin over cricothyroid membrane; may need to extend from mandible to sternum if impalpable anatomy.
  ○ Once skin incised, palpate cricothyroid membrane position and blunt dissect with fingers through subcutaneous tissue until the membrane is readily identifiable.
    ▪ Ignore bleeding until airway is secure.
    ▪ ETT placement usually has a tamponade effect.
  ○ Create a horizontal incision through membrane by dragging scalpel blade from one side to the other; then turn knife through 180 degrees and extend to the other side.
    ▪ The cricothyroid membrane is bound by a “cartilaginous cage” so resistance will be felt at the margins of the membrane when the scalpel blade abuts cartilage.
  ○ Dilate with gloved little finger and palpate tracheal lumen, ideally identifying the cartilage of the posterior wall of the trachea/ cricoid ring.
  ○ Pass bougie alongside little finger into trachea.
  ○ Confirm bougie position with finger, ensuring it passes through membrane.
  ○ Bougie usually holds up at carina <10cm from the skin.
    ▪ do not force if held up as may perforate carina.
  ○ Pass ETT over bougie and intubate trachea.
    ▪ Ensure the ETT balloon is fully deflated and twist ETT as it passes the skin.
    ▪ Only advance the ETT until the balloon is within the airway and no longer visible.
  ○ Ensure ETT is held secure while bougie is removed and ETT is connected to ventilating device.
  ○ Confirm ETT placement with ETCO2 other adjunctive measures.

#### 11. Extra-glottic Devices

*(Author’s note: this section describes devices carried specifically by our program and will need to be adapted to equipment used by the individual HEMS program.)*

##### Overview

As name suggests these devices are positioned ABOVE or AROUND the glottis. There are many devices in this category that utilize different techniques to create a seal around the glottis to provide access for ventilation while providing some degree of airway isolation/protection, but these are not definitive airways. Specifically, 2 different devices are carried. These are as follows:

##### i-Gel

- Functions in the same vein as a laryngeal mask airway,
  ○ Has proprietary technology to mold to a patient’s airway to create a better seal when ventilating,
- Adapted from operating room settings,
- Sizing:
  ○ Size 1, Neonate, 2–5kg
  ○ Size 1.5, Infant, 5–12kg
  ○ Size 2, Small Pediatric, 10–25kg
  ○ Size 2.5, Large Pediatric, 25–35kg
  ○ Size 3, Small Adult, 30–60kg
  ○ Size 4, Medium Adult, 50–90kg
  ○ Size 5, Large Adult, 90+kg[Fig f7-jetem-5-3-c82]

##### King LT

- Dual-balloon device designed to occlude the esophagus and oropharynx allowing for tracheal ventilation.
- Placed blindly but occasionally will achieve blind intubation.
- Sizing:
  ○ Adult 4 Adult 155cm – 180cm, RED
  ○ Adult 5 Large Adult, >180cm, Purple
- Differences in devices:
  ○ i-Gel sizing is based on weight; King LT sizing is height-based in Adults.
  ○ King LT provides an orogastric (OG) port; i-gel has an accessory channel for similar use, but these can be hard to utilize.
  ○ The key benefit to the i-Gel is its shape and design. It is shaped for easy positioning and placement, allowing the user to create a seal without the need for an inflatable balloon.[Fig f8-jetem-5-3-c82]

##### Indications for extra-glottic device placement

- Devices are typically used as a rescue device for the “can’t intubate but can ventilate” situation.
  ○ This can provide a bridge to more definitive airway.
  ○ Provides OG port for decompression of gastric contents.
  ○ Devices can potentially be intubated through or around using fiber optics or other adjuncts.
  ○ Devices may provide a temporary ventilation tool while in the field or during transport if conditions make it difficult or impossible for endotracheal intubation but emergency surgical airway is not indicated.

#### 12. Pelvic Binders

##### Overview

- Pelvic stabilization is an important intervention in the management of severe pelvic trauma.
  ○ Prevents reinjury from pathological pelvic motion.
  ○ Decreases pelvic volume.
  ○ Tamponades bleeding pelvic bones and vessels (usually venous).
  ○ Decreases pain.
- Indicated for patients who have suspected or documented pelvic injuries potentially associated with major hemorrhage.
  ○ Patients with mechanically unstable (disruption of pelvic ring continuity) fractures are the most likely to benefit from pelvic binding.

##### Mechanically Unstable Pelvic Fractures

- Pelvic Binding is indicated for the below fractures:
- AP compression, type II
  ○ Pubic diastasis >2.5 cm.
  ○ anterior sacroiliac (SI) joint disruption (OPEN BOOK).
  ○ Rotationally unstable, vertically stable.
- Lateral compression, type II
  ○ Ipsilateral sacral buckle fractures or ipsilateral horizontal pubic rami fractures.
  ○ ipsilateral iliac wing fracture or posterior SI joint disruption.
  ○ Rotationally unstable, vertically stable.
- Lateral compression, type III
  ○ Type I (described below) or type II (above) on ipsilateral pelvis and external rotation injury, on contralateral pelvis with pubic rami fractures, or disruption of the sacrotuberous and/or sacrospinous ligaments.
  ○ Rotationally unstable, vertically stable.
- AP compression, type III
  ○ Type II (above) plus posterior SI joint disruption.
  ○ Pubic diastasis >4cm.
  ○ Rotationally unstable, vertically unstable.
- Vertical shear
  ○ Vertical pubic rami fractures.
  ○ SI joint disruption +/- adjacent fractures.
  ○ Rotationally unstable, vertically unstable.
- If unknown morphology
  ○ consider empiric pelvic binding in patients with mechanisms and physical exam findings consistent with possible bony pelvic injury as well as hemodynamic instability.

##### Mechanically Stable Pelvic Fractures

- Pelvic binding is NOT indicated:
- AP compression, type I
  ○ Pubic diastasis <2.5 cm.
  ○ Stable.
- Lateral compression, type I
  ○ Ipsilateral sacral buckle fractures.
  ○ ipsilateral horizontal pubic rami fractures (or disruption of symphysis with overlapping pubic bones).
  ○ Stable.

##### Relative Contraindications for Pelvic Binder Placement

- Open pelvic fractures.
- Perineal lacerations.
- Intraabdominal injuries requiring surgery.
- Morbid obesity.
- Burns and severe associated pelvic soft tissues injuries.

##### Placement of Pelvic Binder Placement

*Author’s note: each program will need to provide teaching on the specific style of pelvic binder they use. We utilize manufacturer-produced, trademarked instructions which will be withheld from this publication because of their proprietary nature.*

- General teachings
  ○ Binders should be placed overlying the greater trochanters of the femurs and use this as the point of leverage to “squeeze” the pelvis together.
  ○ Binders should not be loosened once applied, even if hemodynamics improve.
  ○ Skin checks may need to be performed during longer transport because binders place patients at risk of pressure necrosis.

## Appendix G Ventilator Competency

We provide our leaners with an instruction manual which is proprietary to the manufacturer of our ventilators. We encourage programs to do the same and provide learners with the same educational equipment they use to train new staff regarding ventilator use. The teaching provided will vary by the type of ventilator used by the program; however, some suggested topics are provided below to have learners discuss with the medical staff. Learners may have varying levels of comfort with ventilators based on their previous learning experiences as emergency medicine residents. We encourage these teaching sessions to be “hands on” so that learners may practice physically programing settings into a ventilator to familiarize themselves with the placement of buttons and menus.

### Ventilator Discussion Topics

1. The difference between assist control mode and synchronized intermittent mandatory ventilation mode
2. The difference between pressure-based and volume-based ventilation
3. Setting an appropriate tidal volume 4. Changing settings including respiratory rate, tidal volume, positive end expiratory pressure and fraction of inspired oxygen.
4. Meanings of various alarms and how to change alarm settings
5. Different display modes (for ventilators they can display flow, volume, and pressure graphs)
6. Setting up non-invasive positive pressure ventilation (if the ventilator is capable)
7. Setting up an inline nebulizer in the ventilator circuit
8. Setting up inline suction in a ventilator circuit

Once familiarized with the ventilator, learners preform a “test out” with a crew member (see sheet below with performance checklist), demonstrating their basic competency with running the ventilator.

### Performance Checklist

Critical Behaviors[Table t4-jetem-5-3-c82][Table t5-jetem-5-3-c82]

**Table t4-jetem-5-3-c82:**

Completed Ventilator Set Up and Calibration	
Completed Settings, alarms and modes tasks	
Reviewed monitoring and graphics functions	
	

**Table t5-jetem-5-3-c82:**

**Learner:** _________________________________________	**DATE:** ___________
**Evaluator:** _______________________________________	

## Appendix H HEMS Crew Medical Operations

### Section 1: Pre-hospital triage

Pre-hospital medical triage is dependent on the scale of the incident in which a HEMS crew is involved. Typical pre-hospital scenarios requiring triage include motor vehicle collisions with multiple passengers or involving multiple vehicles. These situations, while significant, likely do not require as much coordination within the Incident Command Structure (ICS) enacted by the local authorities, and as such may be more fluid for the HEMS crew. A medical crew may find that they are dispatched to a scene with limited information regarding the medical condition of patients or even the number of patients involved in the incident. In these cases, the crew will need to take the liberty of managing their resources (critical care personnel, equipment, blood products, etc.) Many established triage systems exist for use when there are more patients than available medical providers or equipment, and learners should be given instruction on the established practice of the HEMS operator under which they are training. One such system that can be taught is the Simple Triage And Rapid Treatment (START) method (Figure 1). In other cases, it may make more sense to focus all available energy on the most tenuous patient or the patient whose fate may be most time dependent. This decision-making process should be taught in the context of the typical practice of the HEMS program of which the learner is part.[Fig f9-jetem-5-3-c82]

In cases where a HEMS crew is dispatched as part of a much larger effort, typically in disaster scenarios, it would be expected that a formal ICS would be in place. The HEMS crew would then operate under the direction of the operations director for the ICS, and it is much more likely the crew would need to operate under a triage algorithm such as the START algorithm shown above. If interested, learners should be directed to the “IC-100: Introduction to Incident Command System” course available for free on the Federal Emergency Management Agency website.

### Section 2: Communications with EMS providers

Communicating with EMS providers largely falls into two categories, that of operational and medical information. Typical operational information would include information regarding a description of the established landing zone for the aircraft and report of any potential hazards present on scene (eg, spilled gasoline at a car accident scene). While understanding the process of operational communications between on-scene personnel and HEMS crew is important to the learner, these communications should be performed by an experienced crew member because they directly affect crew safety. Learners can gain experience with performing radio communications with ground EMS by simulating conversations with a fellow HEMS crew member.

Medical communications should be more familiar to a learner because they are similar to EMS handoffs which take place in the emergency department. These communications typically include a brief history of medical symptoms or mechanism of injury, injuries identified, vital signs, and any treatments or interventions performed prior to HEMS crew arrival. Again, this handoff should be performed in the style already established by the HEMS provider, and the learner should be given instruction in this program-specific style.

### Section 3: Communications with transferring or receiving facilities

Communication between HEMS crew members and transferring facilities differs from communications with EMS providers because generally some diagnostics and/or treatments have already been performed. In some instances, the patient has been hospitalized for several days and their disease process is well delineated. Again, this handoff should be performed in the established style of the HEMS program and the learner given instructions specific to that style. In contrast to EMS handoffs, these handoffs may focus more specifically on the reason the patient requires transport and what medical interventions may need to be continued during flight (eg, infusing medications and the current rate of infusion).

When communicating staff at a receiving facility, HEMS crew members often do not need to provide a complete and in-depth history of the patient’s previous hospital course because this information has already been provided to the receiving facility from the transferring facility. Receiving facility staff will, however, be interested to know any significant changes in patient’s vital signs or status during the flight and any interventions or treatments rendered during flight.

## Appendix I Tips for Exposing Learners to Non-Flight HEMS Program Operations

While a significant portion of this curriculum is dedicated to exposing learners to the medical care provided by HEMS crews, there is a significant amount of coordination and management required to make a HEMS program function. We believe it is important for learners to know these structures exist but believe that indepth learning pertaining to management functions exists outside the scope of this curriculum. In our program, we have learners attend the group meetings in charge of guiding aspects of the program (eg, safety, operations, staff education) so that they may understand that these topics are important to ensure a program is operating as safely and effectively as possible. These meetings also expose learners to the processes in which change can be enacted within a program. Since we feel these operations are largely outside the scope of this curriculum, learners participate in the role as an observer but are encouraged to freely ask questions to aid in their understanding.

Approaches to management functions will also vary between programs and will be adapted as such. Below is a list of meetings attended by learners at our program and the intended experiences we hope to expose learners to. We suggest programs implementing this curriculum work to provide learners with similar experiences in whatever way they see fit. If dedicated meetings are not available for learners to attend, some suggestions for implementation are included.

Meetings attended by HEMS rotation learners:

- Critical Care Transport (CCT) education meeting
  ○ Learners should understand that medical crews need to have a full understanding of equipment they are using.
  ○ Most HEMS programs require crew members demonstrate competency with equipment used and any procedures they perform.
    ▪ This requires internal planning and tracking.
  ○ Learners could be asked to lead an education session for a crew competency of which the learner is well experienced, for example, endotracheal intubation.
- Attend CCT performance improvement meeting
  ○ Learners should understand the finite resources available to HEMS programs.
  ○ Review of transports for appropriateness for air transport as well as appropriateness of medical care provided provides valuable information for medical directors.
  ○ Learners could be asked to review a limited number of flight reports to determine if transport by air was indicated and if the medical care provided was appropriate.
- Attend CCT operations meeting
  ○ Learners should understand that any changes made within a program must have approval from stakeholders within the program.
  ○ Functions integral to allowing a program to operate (eg, shift change times, how much staff is needed, which equipment vendors should be used) must all be determined to allow a program to function optimally.
  ○ Learners could discuss with the program’s medical director how change is enacted within a program.
- Attend CCT quality and safety meeting
  ○ Learners should understand that there needs to be a review process in place for equipment malfunctions or misuse as well as any safety incidents that take place during transports.
  ○ Quality and safety meeting objectives align closely with performance improvement but focus more on the non-human factors at play.
  ○ Learners should be exposed to whatever method a program uses to track safety incidents.
- Attend CCT Morbidity and Mortality (M&M) Conference
  ○ This is a meeting that may be specific to our program, but focuses on a “deep dive” into flights with extraordinary circumstances or cases identified by the performance improvement or the quality and safety committees.
  ○ Learners could be asked to review specific safety incidents or quality incidents within a program and asked to identify points of improvement for care.
- Attend all critical care staff monthly meetings
  ○ A general meeting which demonstrates how information is disseminated among a program’s employees.
  ○ Allows learners to meet a larger proportion of program staff.
  ○ Learners could meet with the program’s medical director to discuss how a program circulates new information and program updates among staff.
